# Acute Hepatitis E-Associated Guillain-Barré Syndrome

**DOI:** 10.7759/cureus.48778

**Published:** 2023-11-14

**Authors:** Ricardo A Rodrigues, Miguel Sequeira, Francisco Barros, Telma Alves, João Gonçalves

**Affiliations:** 1 Internal Medicine, University Hospital Center of Coimbra, Coimbra, PRT; 2 Intensive Medicine, University Hospital Center of Coimbra, Coimbra, PRT; 3 Neurology, University Hospital Center of Coimbra, Coimbra, PRT

**Keywords:** hev, polyneuropathy, demyelinating polyneuropathy, acute viral hepatitis, auto-immune, guillain barre’s syndrome (gbs), hepatitis e virus

## Abstract

Guillain-Barré syndrome (GBS) is a rare autoimmune disorder of the peripheral nervous system that causes progressive weakness and sensory disturbances, usually following an infection or immunization. It has been associated with multiple causes, including bacterial and viral infections. Hepatitis E virus (HEV) is a common cause of acute viral hepatitis that can rarely develop neurological complications. We report a case of a 72-year-old man who developed GBS secondary to an acute HEV infection. He presented with numbness and weakness of the lower limbs that rapidly evolved into respiratory failure requiring mechanical ventilation and intravenous immunoglobulin therapy (IVIg). This case adds to the literature on the association between HEV infection and GBS and the importance of early detection of this rapidly progressive condition.

## Introduction

Guillain-Barré syndrome (GBS) is a rare disorder of the peripheral nervous system characterized by progressive weakness and sensory disturbances, usually following an infection or immunization [1]. Hepatitis E virus (HEV) is a common cause of acute viral hepatitis worldwide, transmitted mainly through contaminated water or food. Although HEV infection is usually self-limiting and asymptomatic, it can occasionally trigger GBS as a post-infectious complication [2-4]. Here, we report a case of a 72-year-old man who developed GBS secondary to an acute HEV infection.

## Case presentation

A 72-year-old male presented to the emergency department with symmetrical numbness and weakness in both lower limbs. He experienced difficulty walking, which began upon awakening and rapidly progressed to involve both upper limbs symmetrically. He had been discharged the day prior after an elective cataract surgery. The patient's past medical history was relevant for hypertension, type 2 diabetes mellitus with no known microvascular complications, and hypercholesterolemia, and the patient was on Atorvastatin, Azilsartan, Chlorthalidone, Metformin, and Dapagliflozin.

Neurological examination revealed a relatively symmetric paraparesis with grade 4/5 strength on lower limbs and diminished sensation in a stocking-glove distribution. Upper limb strength was unaltered. There were no changes in cranial nerves and no signs of meningeal irritation. The remaining physical examination was unremarkable.

Laboratory tests showed elevated liver enzymes, and positive serology for HEV with a high viral load (Table 1). Hepatitis A, B, C, and D serologies, as well as other viral serologies and autoimmune studies, were negative. Abdominal ultrasound showed slightly increased echogenicity, suggestive of steatohepatitis. 

**Table 1:** Laboratory test results and evolution of the metabolic panel.

	At admission	At discharge	Reference ranges
Metabolic panel			
Lactate dehydrogenase (LDH)	954 U/L	191 U/L	<248 U/L
Aspartate aminotransferase (AST)	1,834 U/L	37 U/L	<35 U/L
Alanine aminotransferase (ALT)	2,139 U/L	62 U/L	<45 U/L
Alkaline phosphatase (ALP)	119 U/L	138 U/L	30-120 U/L
Gamma-glutamyltransferase (GGT)	527 U/L	386 U/L	<55 U/L
Total bilirubin	1.2 mg/dL	0.7 mg/dL	0.2-1.2 mg/dL
Hepatitis E serology and viral load			
Anti-HEV (IgG)	0.66 UI/mL	-	<0.3 UI/mL
Anti-HEV (IgM)	3.63 UI/mL	-	<1.0 UI/mL
HEV viral load	1,977,133 UI/mL	-	
Cerebral spinal fluid laboratory results			
Proteins	76 mg/dL	-	15-40 mg/dL
Glucose	107 mg/dL	-	40-70 mg/dL
Lactate	2.62 mml/L	-	<2 mmol/L
Leucocytes	<3 mm^-3^	-	<3 mm^-3^

The patient was admitted to the ward; however, he rapidly deteriorated with worsening quadriparesis, hyporeflexia, dysphagia, and dyspnea. A lumbar puncture was performed, showing albuminocytologic dissociation with increased proteins and a normal cell count (Table 1). Electromyography showed decreased conduction velocities suggestive of generalized demyelinating polyneuropathy. The patient was diagnosed with GBS and started on IVIg, 0.4 g/kg per day. Despite these measures, clinical deterioration continued, and the patient developed respiratory failure, requiring endotracheal intubation and mechanical ventilation. He was transferred to the intensive care unit for further management.

The patient received a five-day course of IVIg, which resulted in gradual improvement of his neurological status. He was successfully extubated on day 14 and transferred to the neurology ward. Liver enzymes normalized with supportive care, and liver function was preserved throughout the illness. At discharge, the patient maintained areflexia with mild residual weakness in the proximal muscles of the lower and upper limbs and no changes in sensation. He was referred to a rehabilitation clinic for further recovery.

## Discussion

This case illustrates a rare but serious complication of acute HEV infection, namely, GBS [5]. GBS is an autoimmune disorder that affects the peripheral nerves, causing progressive weakness, sensory disturbances, and sometimes respiratory failure. The exact pathogenesis of GBS is not fully understood; however, it is believed to be triggered by molecular mimicry between microbial antigens and nerve components, leading to an aberrant immune response. GBS is usually preceded by an infectious disease (mainly respiratory or gastrointestinal), with several microorganisms being linked to the onset of disease, such as Campylobacter jejuni, Cytomegalovirus, Epstein-Barr virus, and Zika virus [5,6]. Although rare, it has also been associated with acute hepatitis infections, with cases reported in the literature for GBS following acute viral hepatitis A, B, C, D, and E [2-4].

HEV infection is endemic in many developing countries, where it is transmitted mainly through the fecal-oral route via contaminated water or food. However, HEV infection is also increasingly recognized in developed countries, where it is transmitted mainly through zoonotic sources, such as pigs and wild boars [7]. HEV infection can cause acute hepatitis, chronic hepatitis in immunocompromised patients, and extrahepatic manifestations, such as neurological, renal, hematological, and rheumatological disorders. There are several neurological manifestations associated with HEV, including but not limited to GBS, Bell’s palsy, meningitis, encephalitis, and myelitis. Neurological manifestations affect roughly 5.5% of patients following acute or chronic HEV infection [7,8].

The diagnosis of HEV-associated GBS relies on the detection of HEV RNA or antibodies in serum or cerebrospinal fluid (CSF), along with the clinical, laboratory, and electrophysiological features of GBS [2,4]. CSF examination will show increased protein levels with normal leukocytes (albuminocytologic dissociation). Electrophysiological studies will typically show peripheral nerve conduction slowing or blocking and prolonged distal motor latency, although these findings can be normal in the early stages [5,6].

The treatment of HEV-associated GBS is similar to that of idiopathic GBS, consisting of immunomodulatory therapies, such as IVIg or plasma exchange. The prognosis of HEV-associated GBS is generally good, with most patients recovering fully or partially within six months. However, some patients may have residual neurological deficits or require prolonged mechanical ventilation [4-6].

## Conclusions

We report a rare case of GBS secondary to an acute HEV infection, which presented with severe neurological and hepatic manifestations. The patient responded well to IVIg therapy and supportive care and had a favorable outcome. This case underscores the possibility of GBS as a complication of various infections and the need for clinicians to pay attention to rapidly worsening signs and symptoms that may indicate this condition. Early diagnosis and treatment of GBS can prevent life-threatening complications and improve prognosis. This case also adds to the growing literature on the association between HEV infection and various neurological disorders, which may have important implications for the epidemiology, pathogenesis, and management of these conditions.
